# Executive Summary of the 2024 Philippine Clinical Practice Guidelines on the Diagnosis and Management of Acute Severe Blood Pressure Elevation

**DOI:** 10.1111/jch.70199

**Published:** 2026-01-30

**Authors:** Deborah Ignacia D. Ona, Felix Eduardo Punzalan, Raymond V. Oliva, Ma. Sergia Fatima Sucaldito, Noel Espallardo, Richard Santos, Hannah Almenario, Karl Murillo, Valentin Dones, Elmer Jasper Llanes, Lourdes Ella Santos, Maria Cristina San Jose, Alyssa Samantha Fusingan‐Peralta, Rommel Bataclan, Maria Katrina Mata, Pauline Convocar, Aurelia Leus, Gilbert Vilela

**Affiliations:** ^1^ Division of Cardiovascular Medicine University of the Philippines College of Medicine Manila‐Philippine General Hospital Manila Philippines; ^2^ St. Luke's Medical Center Quezon City Philippines; ^3^ Division of Adult Medicine Department of Medicine University of the Philippines College of Medicine Manila‐ Philippine General Hospital Manila Philippines; ^4^ Samar Island Institute of Medicine Samar State University Catbalogan Philippines; ^5^ Department of Emergency Medicine and Acute Care Pasig City General Hospital Pasig Philippines; ^6^ College of Rehabilitation Sciences University of Santo Tomas Manila Philippines; ^7^ Department of Medicine Cardinal Santos Medical Center San Juan Philippines; ^8^ Department of Neurosciences University of the Philippines College of Medicine Manila‐ Philippine General Hospital Manila Philippines; ^9^ Department of Medicine University of the East Ramon Magsaysay Medical Center Quezon City Philippines; ^10^ Department of Emergency Medicine Manila Doctor's Hospital Manila Philippines; ^11^ Department of Emergency Medicine Southern Philippines Medical Center Davao City Philippines; ^12^ Department of Emergency Medicine Corazon Locsin Montelibano Memorial Regional Hospital Bacolod City Negros Occidental Philippines; ^13^ Makati Medical Center Makati City Philippines; ^14^ Philippine Heart Center Quezon City Philippines

**Keywords:** acute severe hypertension, cardiovascular disease, guidelines, hypertensive emergency, hypertensive urgency

## Abstract

A recent survey in the Philippines, PRESYON‐4, showed increasing prevalence of hypertension from 22% in the 1990s to 37% in 2021, of which only 52% were aware of their diagnosis. While rates of treatment and adherence were 68% and 86%, respectively, the rate of BP control was low at 37%. Furthermore, there remained a high degree of unawareness regarding hypertension, its role in CV morbidity and mortality, and how it can be optimally managed. In particular, there is a knowledge gap in the diagnostic approach and management of severe acute elevations in blood pressure. In response to this, the Philippine Society of Hypertension, Philippine Heart Association, and multiple experts from various sectors worked together to develop the 2024 Clinical Practice Guideline on the Diagnosis and Management of Severe Blood Pressure Elevation. The CPG provides eleven (11) recommendations and four (4) best practice statements addressing key clinical questions on the diagnosis and management of severe BP elevation. The guideline development process adhered to the GRADE approach through the Evidence to Decision (EtD2) framework, including the identification of critical questions and outcomes, retrieval of current evidence, appraisal and synthesis of the evidence, and formulation of draft recommendations. A multisectoral consensus panel (CP) was convened to discuss values, preferences, and socioeconomic impact and finalize the strength of the recommendations. The CPG is intended to be used by general practitioners, specialists, family physicians, allied health professionals, emergency medical personnel, and healthcare workers who may encounter adult patients with hypertension, whether in the inpatient or outpatient setting.

## 1 Introduction

Atherosclerotic cardiovascular disease (ASCVD) is one of the leading causes of death in the world. In the Philippines, ischemic heart disease (IHD) remains the most common cause of death, while cerebrovascular disease or stroke is the third most common [1]. ASCVD, including IHD and stroke, arises as end‐organ complications of prevalent and preventable non‐communicable diseases (NCD) and their risk factors. The most common of these NCDs—hypertension—remains a prime modifiable risk factor in the progression of ASCVD, and its treatment provides an important opportunity for primary and secondary CV prevention. Optimal management of hypertension has been unequivocally proven to reduce the morbidity and mortality associated with ASCVD [2].

The most recent survey on hypertension in the Philippines, PRESYON‐4, showed an increasing trend of prevalence from 22% in the 1990s to 37% in 2021 [3]. Of these, only 52% were aware of their diagnosis. While rates of treatment and adherence were found to be 68% and 86%, respectively, the rate of BP control was low at 37%. Furthermore, there remained a high degree of unawareness regarding hypertension, its role in CV morbidity and mortality, and how it can be optimally managed. These findings confirmed that hypertension remains a priority problem for Filipinos and that its control is a low‐hanging fruit that will have far‐reaching beneficial effects for Filipinos in decades to come.

Given the J‐shaped correlation of hypertension with ASCVD [4, 5, 6], much attention has been paid in particular to the management of acute and severe elevations in BP among patients with and without known hypertension. The 2025 ACC/AHA guideline for hypertension among adults defined systolic blood pressure (BP) >180 mmHg and/or diastolic BP > 120 mmHg associated with evidence of acute target organ damage as “hypertensive emergency” [7]. The most recent european society of cardiology (ESC) guidelines define a hypertensive emergency similarly as a BP of ≥ 180/110 mmHg associated with acute hypertension‐mediated organ damage (HMOD) [8]. Similar elevations in BP but without acute target organ damage have been labeled “hypertensive urgency” by various authors and mentioned in several guidelines [9]. Both hypertensive urgency and hypertensive emergency were grouped under the unofficial umbrella term “hypertensive crisis,” a concept that has recently evolved to a more inclusive term, “acute severe BP elevation”.

Hypertensive emergency has been linked with higher rates of admission and mortality in observational studies [10]. However, evidence on the management of hypertensive emergencies is mixed [11, 12]; it cannot be ascertained if the benefit observed is attributable to the treatment of the severe BP elevation or the management of the underlying condition, such as acute coronary syndrome (ACS) or stroke, which often also includes lowering BP [13, 14]. In contrast, there is no evidence that treating hypertensive urgency in the emergency setting offers any distinct benefit in reducing major adverse cardiovascular events (MACE) compared to routine outpatient management of chronic hypertension. Hence, additional treatment beyond outpatient medication introduction or modification and tailored follow‐up is not recommended by the AHA or ESC [7, 8].

Due to the lack of evidence, the terms “hypertensive crisis” and “hypertensive urgency” have been endorsed for retirement. [14, 15] Furthermore, the AHA 2024 Scientific Statement on the Management of Hypertension in the Acute Care Setting, recommends the transition of the terms “hypertensive crisis” and “hypertensive urgency” to “markedly elevated BP” and “asymptomatic markedly elevated BP”, respectively. In alignment with this terminology shift, we will be referring to the concept previously known as “hypertensive urgency” as “acute severe hypertension.” For the concept described by the term “hypertensive crisis,” we will use the term “acute severe BP elevation” since the conditions comprising it are sufficiently different and warrant varying approaches to management. The term “hypertensive emergency” is retained throughout the guideline as evidence supports emergent treatment for this group of conditions.

Despite this important shift in terminology, we acknowledge that most literature on these concepts still uses the terms “hypertensive urgency” and “hypertensive crisis,” and thus we retained their use only for the search strategies of our literature reviews. Medical literature has yielded a mix of evidence on the optimal approach to managing patients with acute severe BP elevation; hence, the Task Force (TF) created this guideline to evaluate the best available evidence and inform healthcare providers on the critical aspects of the diagnosis and management of these conditions.

## 2 Methodology

Experts in the fields of hypertension, general practice, internal medicine, family medicine, cardiology, emergency medicine, public health, and the academe, including a patient advocate, were assembled to comprise the TF for this guideline. The guideline development process adhered to the Grades of Recommendation, Assessment, Development, and Evaluation (GRADE) approach [16]. The Steering Committee (SC) convened, identified the key clinical questions, and specified the population, intervention, and outcomes for each question. Systematic searches for relevant studies to answer the clinical questions were carried out by qualified evidence review experts (EREs) vetted by the SC.

The literature search was conducted through search engines, including PubMed, Scopus, Medline, Google Scholar, and Cochrane Library, and was further widened by searching through clinical trial registers and investigating cross‐references. The cut‐off date of the search was February 1, 2024. Unpublished data were also retrieved whenever possible. The EREs synthesized the evidence into standardized evidence profiles and evidence‐to‐decision frameworks. These were presented to a consensus panel (CP), including a patient advocate, who discussed the socioeconomic, applicability, and feasibility considerations. Panel members voted on the direction and strength of recommendation through the Modified Delphi technique.

## 3 2024 Clinical Practice Guidelines on Acute Severe Blood Pressure Elevation

### 3.1 Best Practice Statement 1

- Among patients with SBP equal to or greater than 180 mmHg or DBP equal to or greater than 120 mmHg without signs and symptoms of hypertension‐mediated organ damage, we recommend the term “acute severe hypertension” instead of “hypertensive urgency.”

The TF tackled the issue of the evolving terminology for severe BP elevation and concurred with the paradigm shift away from the terms “hypertensive crisis” and “hypertensive urgency.” This shift is due to evidence of a lack of benefit from urgent treatment of patients with so‐called hypertensive “urgency”, in addition to the possible harms of rapidly lowering BP among these patients [7, 14, 15]. This led to the formulation of this best practice statement, which labels SBP equal to or greater than 180 mmHg or DBP equal to or greater than 120 mmHg without signs and symptoms of hypertension‐mediated organ damage as “acute severe hypertension.”

### 3.2 Clinical Question 1

Among hypertensive patients, what are the focused clinical history and physical exam (PE) findings to consider the patient as acute severe hypertension or hypertensive emergency?

### 3.3 Statement 1

- Among adult patients presenting with an elevated blood pressure of SBP ≥ 180 mmHg or DBP ≥ 120 mmHg, we recommend eliciting the presence of at least one of the main symptoms:
- focal neurologic complaints
- visual impairment
- dyspnea
- chest pain
- headache
  when deciding to evaluate further for the diagnosis of hypertensive emergency.
  (certainty of evidence very low, strength of recommendation strong)

#### 3.3.1 Evidence From Literature

A systematic review and meta‐analysis of eight studies (five prospective and three retrospective studies) with a total of 1 970 patients with hypertensive emergency and 4 983 patients with hypertensive urgency investigated the prevalence of both disease entities, predictive values of specific symptoms, degree of BP elevation, and risk factors, as well as the relative frequency of acute HMOD [17]. A second systematic review and meta‐analysis analyzed 19 studies looking into non‐modifiable (age, sex, and ethnicity) and modifiable factors (socioeconomic status [SES], adherence to medical therapy, comorbidities, and substance abuse) of adult hypertensive patients and their impact on the likelihood of hypertensive urgency and emergency [18]. A retrospective cohort of 718 patients presenting with elevated BP at the ED of a hospital in Turin, Italy, looked into the diagnostic accuracy of specific presenting symptoms associated with the diagnosis of hypertensive emergency [19]. Specifically, the diagnostic performance of an algorithm proposed by Van der Born et al. and endorsed by the ESC in 2019 was evaluated [15].

Upon presentation at the ED, there was no significant difference in SBP values among patients with hypertensive emergency and urgency (MD 1.4 mmHg; 95% CI −0.8 to 3.6), while the DBP values were found to be slightly higher for hypertensive emergency (MD 2.3 mmHg; 95% CI 0.3 to 4.3) [17]. For the presenting symptoms, a study showed that the algorithm endorsed in 2019 by the ESC for use in diagnosing true hypertensive emergency has a diagnostic accuracy of 64%, sensitivity of 94%, specificity of 60%, negative predictive value (NPV) of 99%, and a positive predictive value (PPV) of 23%. A strong association was noted between the main symptoms and hypertensive emergency after logistic regression analysis (OR: 18.315, 95% CI 7.82 to 42.9) [19]. Among the symptoms, focal neurological signs had the highest PPV of 51.6%, followed by dyspnea at 25.8%, visual impairment at 20%, chest pain at 11.3%, and headache at 4.4%.

The presence of comorbidities such as coronary artery disease (OR 1.654, 95% CI 1.232 to 2.222), congestive heart failure, cerebrovascular disease (OR 1.769, 95% CI 1.218 to 2.571), and chronic kidney disease (OR 2.899,95% CI 1.32 to 6.364) was associated with a higher risk of hypertensive crises in general [18]. In the same study, hypertensive emergency was more common in men (OR 1.390, 95% CI 1.2071 to 1.601), older patients (MD 5.282, 95% CI 0.624 to 3.461), and those with diabetes (OR 1.723, 95% CI 1.485 to 2.000) and hyperlipidemia (OR 2.028, 95% CI 1.642 to 2.505). In contrast, non‐adherence to antihypertensive medication and unawareness of the hypertension diagnosis did not increase the risk of hypertensive emergency.

#### 3.3.2 Consensus Panel Synthesis

Regardless of whether a patient has a prior diagnosis of hypertension, screening for the above symptoms is strongly recommended due to their high specificity for hypertensive emergency, the benefits of rapidly identifying hypertensive emergency, and the importance of promptly instituting life‐saving management. This recommendation covers any patient with SBP ≥ 180 mmHg or DBP ≥ 110 mmHg regardless of known hypertension status, given: (1) the high prevalence of hypertension unawareness in the Philippines estimated at 48% of persons with hypertension in the PRESYON‐4 survey, (2) representation of both aware and unaware populations in the best available evidence, and (3) the crucial implications of quickly clinching the diagnosis of hypertensive emergency. Notably, there were no significant differences in presenting BP at the emergency department (ED) among patients with hypertensive emergency or acute severe hypertension; hence, the degree of BP elevation is not a reliable indicator for differentiating the two conditions.

## 4 Acute Severe Hypertension

### 4.1 Clinical Question 2

Among patients with acute severe hypertension, what are the indications for referral to a hospital facility?

### 4.2 Statement 2

- Among patients with acute severe hypertension, we suggest screening for signs and symptoms of hypertension‐mediated organ damage to rule out hypertensive emergency, which will warrant referral to a hospital.
- Older age (> 60 years old), male sex, presence of chronic kidney disease, and proteinuria are risk factors for poor outcome among patients with acute severe hypertension, which necessitate close monitoring but can be managed in the outpatient setting.
  (certainty of evidence: low, strength of recommendation: weak)

#### 4.2.1 Evidence From Literature

There were no studies that directly answered the clinical question on the indications for referral to a hospital for patients with acute severe hypertension. Although no direct evidence exists, patients in the outpatient setting with acute severe hypertension and with symptoms such as headache, dizziness, chest pain, shortness of breath, vomiting, or blurred vision warrant further evaluation in the hospital to rule out the presence of HMOD as mentioned in Statement 1.

There were no studies to show that acute severe hypertension is associated with an acute elevation in cardiovascular risk. There were two observational studies that looked into all‐cause mortality and major adverse cardiovascular events (MACE) rates among patients with acute severe hypertension. Factors that were associated with a higher incidence of 3‐year mortality were older age (> 60 years old), male sex, presence of chronic kidney disease, and proteinuria [20]. However, in another observational study, patients with acute severe hypertension who were referred to a hospital emergency department showed no difference in MACE after 6 months compared to those who were sent home from the clinic [14].

#### 4.2.2 Consensus Panel Synthesis

During the evidence review, there was a lack of controlled clinical trials investigating the benefit of referring to urgent care for patients with acute severe hypertension, with nonrandomized studies showing no benefit in this population. Among patients with acute severe hypertension, there was no difference in major adverse cardiovascular events (MACE) between those who were sent home compared to those referred to a hospital for further management. Therefore, the importance of eliciting symptoms of hypertension‐mediated organ damage as recommended in Statement 2 is mandatory to differentiate acute severe hypertension from hypertensive emergency, which would warrant prompt referral to a hospital. Furthermore, factors associated with 3‐year mortality, such as older age (> 60 years old), male sex, the presence of chronic kidney disease, and proteinuria, were identified.

### 4.3 Clinical Question 3

Among patients with acute severe hypertension, what is the effect of antihypertensive drugs in reducing blood pressure and other cardiovascular clinical outcomes (mortality, stroke, and MI)?

### 4.4 Statement 3

- Among adult patients with acute severe hypertension whose BP does not respond after two hours of rest, we suggest the use of oral antihypertensive medications* to decrease BP.
  (certainty of evidence: very low, strength of recommendation: weak)
  *

#### 4.4.1 Evidence From Literature

Five studies (1 RCT, 3 retrospective cohort, 1 retrospective descriptive) involving a total of 24 338 patients were identified. All studies were primarily done in Asia—one RCT in South Korea [21], two retrospective cohort studies done in Thailand [22, 23], one observational study done in Thailand [24], and one retrospective cohort study done in China [11]. Most studies included only patients with acute severe hypertension (formerly “hypertensive urgency”), except for one study, which had patients with hypertensive crisis not further subdivided into urgency or emergency [11]. All studies were done in the ED and assessed the effect of administering antihypertensives.

Outcomes measured included BP reduction [21, 22, 24], MACE including stroke, myocardial infarction, and aortic dissection at 1 and 7 days [22], 7‐day, 30‐day, and 60‐day hospital revisits, CV mortality at 1, 3, and 5 years, incidence of stroke at 1, 3, and 5 years, [11] and adverse events [22, 23, 24]. The single‐center, randomized controlled trial evaluated the efficacy of resting and antihypertensive medications among 138 patients with hypertensive urgency. The study investigated the effect of 2 h of rest compared to administering antihypertensive medications on the BP and the rate of BP lowering. They found that the number of patients who achieved a target mean BP reduction of 10%–35% was 68.5% in the resting group and 69.1% in the antihypertensive therapy arm, showing that there was no significant difference between the two groups (*p* = 0.775). The mean changes in the SBP and DBP of both groups (rest vs. antihypertensive drugs) were also not significantly different (−32.2 ± 23.3 vs. −32.8 ± 24.5, *p* = 0.065, and −21.2 ± 18.5 vs. −18.6 ± 17.5, *p* = 0.352, respectively). Of the 138 participants, 99 (49 in the resting group and 50 in the antihypertensive therapy group) were subsequently followed up 24 h and 7 days after ED admission. The SBP and DBP were also not significantly different between the two groups at 24 h (*p* = 0.459 and *p* = 0.512, respectively) and at 7 days (*p* = 0.397 and *p* = 0.293, respectively) [21].

In a 2020 retrospective cohort study conducted by Sricharoen et al., they compared the revisit rates at 1 and 7 days, MACE (stroke, myocardial infarction, congestive heart failure, and aortic dissection) at 1 and 7 days, reduction of BP, and BP control within 2 weeks among patients with hypertensive urgency who were treated with oral antihypertensives (*n* = 298) and those who were not (*n* = 394). Results showed that between the two groups, there was no significant difference in 1‐day revisits [adjusted OR 0.66 (CI: 0.33–1.35), *p* = 0.256], 7‐day revisits [adjusted OR 0.99 (CI: 0.70–1.41), *p* = 0.964], MACE [adjusted OR 0.23 (CI: 0.01–4.18), *p* = 0.321], and BP control in 2 weeks [adjusted OR 0.76 (CI: 0.45–1.30), *p* = 0.319]. Among the secondary outcomes in the study, the reductions of SBP and DBP were also not significantly different in those who were treated with antihypertensives and those who were not (*p* = 0.180 and *p* = 0.589, respectively). However, these outcomes measured only short‐term risks and may not reflect the long‐term risks or benefits of antihypertensive therapy [22].

A larger retrospective cohort study that involved 22,906 adult patients also showed minimal benefit in treating patients with severe hypertension with antihypertensives. The study by Lin et al. in 2021 (*n* = 22 906) examined patients who were treated in the ED and received pharmacologic BP intervention (*n* = 6364) and those who had non‐pharmacologic BP intervention (*n* = 16 542). The study showed an 11% reduction in hospital revisits at 30 days (CI: 0.82–0.97) and at 60 days (CI: 0.82–0.96) in the pharmacologic BP intervention arm. Although there was a 6% reduction in hospital revisit at 7 days among those treated with antihypertensives, it was not clinically significant [adjusted hazard ratio (aHR) 0.94 (CI: 0.84–1.06)]. There was also no clinically significant reduction in CV mortality at 1 year [aHR 0.97 (CI: 0.67–1.41)], 3 years [aHR 0.95 (CI: 0.75–1.19)], and at 5 years [aHR 0.89 (CI: 0.74–1.08)] among patients treated. Both groups had equivalent incidence of stroke at 1 year [aHR 0.84 (CI: 0.59–1.19)], 3 years [aHR 0.84 (CI: 0.66‐1.08)], and 5 years [aHR 0.81 (CI: 0.65–1.01)]. However, the same study also demonstrated that treatment with antihypertensives during an episode of severe hypertension may be more beneficial among those with polypharmacy, as evidenced by a 17% (CI: 0.75–0.93) and 22% (CI: 0.72–0.86) reduction in the CV mortality risk at 3 and 5 years, respectively. It is of note that the study did not differentiate whether the patients presented with acute severe hypertension or hypertensive emergency [11].

In terms of safety, a retrospective cohort study done in 2018 by Kotruchin, et al. determined the prevalence of acute severe hypertension in an ED in Thailand. In the study of 451 patients who presented with acute severe hypertension, 408 were given antihypertensive medications, and there were no noted adverse events upon administration, regardless of the number of antihypertensives given or the combination of antihypertensives. Similarly, in the aforementioned study conducted by Sricharoen et al., adverse outcomes from antihypertensive medications (hypotension) at 1‐day and 7‐day duration found no incident of hypotension secondary to antihypertensives [23]. Similar findings were observed in a retrospective descriptive study by Sruamsiri et al., wherein medical records of patients at the ED with severe hypertension (SBP > 180 and/or DBP > 120) without end‐organ damage (*n* = 151) were retrieved. Patients were given oral antihypertensives (captopril, hydralazine, nifedipine, or amlodipine), and repeat BP measurements were taken at 30 or 60 min, depending on the drug given. No adverse events were noted throughout the emergency visit; however, the study did not disclose the duration of observation of these patients. [24]

#### 4.4.2 Consensus Panel Synthesis

Among adults with acute severe hypertension, studies showed that the administration of antihypertensive drugs within 15–30 min was not significantly different from providing two hours of rest in terms of reducing SBP and DBP at the ED. Furthermore, the incidence of cardiovascular mortality, stroke, and MACE was also not significantly different at 2 h. While there was no difference in CV outcomes, the administration of oral antihypertensive drugs within 15–30 min was well‐tolerated, and there was no increase in adverse events, such as hypotension. There was also a lack of direct evidence on the benefit/harm of giving antihypertensive medications emergently within 15–30 min compared to routine initiation and/or intensification of medication on an outpatient basis. Given unclear benefits and risks of aggressive BP reduction in terms of additional monitoring, medical tests, medications, and unnecessary referrals, the TF suggests allowing 2 h of rest for these patients, followed by clinical reassessment. If the patient continues to have acute severe hypertension after this period and in the absence of new‐onset signs of HMOD during reassessment, initiation of new or intensification of existing oral antihypertensive medications is suggested. The guideline highlights the need for individualizing evaluation for the cause of the BP elevation, tailoring treatment, and sharing decision‐making when deciding to emergently treat patients with acute severe hypertension, given the heterogeneous clinical characteristics of these patients.

### 4.5 Best Practice Statement 2

- Among adult patients with acute severe hypertension, we suggest out‐of‐office BP monitoring with home BP or 24 h ambulatory BP monitoring and follow‐up within 1 week.

Given the lack of evidence suggesting the benefit or harm of providing urgent antihypertensive therapy beyond the 2 h time frame, a Best Practice Statement was drafted to guide follow‐up care. The TF suggests out‐of‐office BP monitoring with a home BP monitor or 24 h ambulatory BP monitoring and a close follow‐up within 1 week.

### 4.6 Clinical Question 4

Among patients with acute severe hypertension, should sublingual or oral medications be used to reduce blood pressure?

### 4.7 Statement 4

- Among adult patients with acute severe hypertension, we recommend the use of oral antihypertensive medications over sublingual antihypertensive medications to lower BP.
  (certainty of evidence: very low, strength of recommendation: strong)

#### 4.7.1 Evidence From Literature

We included 8 RCTs on efficacy from two systematic reviews by Campos et al. and Cherney and Strauss [25, 26]. Overall, there were 261 participants in the treatment group and 264 participants in the control group. Interventions included calcium channel blockers (CCBs) like nifedipine and nicardipine, beta‐blockers like labetalol, and alpha agonists like clonidine [27, 28, 29, 30, 31]. Atkin et al. investigated the effects of labetalol 200 mg versus sublingual clonidine 0.1 mg at varying regimens. The study found that after 4 h, the mean SBP was significantly lower in the clonidine group (149 mmHg, SD 7.2) compared to the labetalol group (166 mmHg, SD 4.7) (*p* < 0.05) [30]. Similarly, Jaker et al. compared the effects of nifedipine 20 mg against sublingual clonidine 0.1 mg on both SBP and DBP. After 1 h, the mean SBP in the nifedipine group was lower at 152 mmHg (SD 6.2) compared to the clonidine group, with a mean SBP of 185 mmHg (SD 3.6) (*p* < 0.05). At 1 h, patients who received nifedipine also had a significantly lower mean DBP of 100 mmHg (SD 3.2) compared to 120 mmHg (SD 2.6) in the clonidine group (*p* < 0.01). At the 2 h mark, the mean DBP in the nifedipine group was 101 mmHg (SD 2.9) versus 113 mmHg (SD 2.6) in the clonidine group (*p* < 0.05) [31].

Studies by Komsuoglu et al., Maleki et al., and Kaya et al. predominantly utilized sublingual calcium channel blockers (CCBs) such as nifedipine and nicardipine compared to oral angiotensin‐converting enzyme inhibitors (ACEi) like captopril [32, 33, 34]. At 1 to 4 h, the reported mean reductions in SBP and DBP were not statistically significant (*p* > 0.05). In comparing the two routes of administration, sublingual captopril 25 mg exhibited non‐significant reductions in SBP and DBP at 1 h compared to oral captopril 25 mg [32]. In conclusion, oral antihypertensives appear to be as effective as sublingual medications in reducing BP over longer durations in inpatient settings. While sublingual antihypertensives may offer rapid relief, they generally do not show statistically significant reductions when compared with other antihypertensives in the short term.

#### 4.7.2 Consensus Panel Synthesis

There was insufficient evidence on the direct comparison of sublingual and oral medications in terms of the degree and rate of BP lowering among patients with acute severe hypertension. However, studies exploring the benefit of lowering BP urgently over minutes to hours, as would occur with administration of sublingual medication, found no benefit compared to the gradual lowering associated with routine initiation/titration of outpatient medications. In addition, anecdotal reports discussed harms and adverse events associated with lowering BP urgently, such as watershed infarcts. Sublingual medications, which have shorter half‐lives compared to oral medications, may also predispose to greater BP variability and poorer CV outcomes. Due to the limited predictability of response to sublingual medications, there is a greater requirement for patient education when prescribing them compared to oral medications. In contrast, oral medications produce less BP variability and add less complexity to the medication regimen, which may promote long‐term adherence. Due to these considerations, the TF strongly recommends using oral antihypertensive agents to control BP among patients with acute severe hypertension.

### 4.8 Clinical Question 5

Among patients with acute severe hypertension, what is the effect of gradual lowering (24 h) versus immediate lowering (minutes to hours) of blood pressure in reducing cardiovascular mortality, stroke, and MI?

### 4.9 Statement 5

- Among adult patients with acute severe hypertension, we recommend a gradual reduction of BP over 24 to 48 h over immediate lowering to normal levels to reduce all‐cause mortality, MACE, and ED re‐visits.
  (certainty of evidence: very low, strength of recommendation: strong)

#### 4.9.1 Evidence From Literature

Randomized controlled trials investigating this topic are currently lacking. Two observational studies examined the outcomes of patients presenting with hypertensive urgency, comparing those who received immediate intervention in the ED to those who did not. A single‐center study by Levy et al. included 1016 patients, predominantly African Americans, with hypertensive urgency at the ED, of whom 435 (42.8%) received antihypertensive therapy. The primary medication administered was oral clonidine (88.5%), with other treatments including IV labetalol (10.1%), hydralazine (7.6%), enalapril (1.6%), and metoprolol (0.5%). The interim BP reduction was nearly double for patients who were given an antihypertensive agent, with a drop in systolic BP of 39.8 mmHg (vs. 19.7 mmHg) and diastolic BP of 21.1 mmHg (vs. 10.0 mmHg). ED revisits at 24 h and 30 days, hospital admission for hypertension‐related complications at 24 h and 7 days were similar whether they received antihypertensive therapy or not. All‐cause mortality was low with no difference at 30 days in treated and untreated groups (0.2% vs. 0.2%; difference of 0, 95% CI −1.1 to 0.8) or 1 year (2.1% vs. 1.6%; difference of −0.5, 95% CI −2.5 to 1.2). The occurrence of adverse events was minimal, and death rates were low in both treated and untreated patients. Overall, the study did not demonstrate clear benefit nor harm from not treating elevated BP at the ED [35].

In a study conducted at Ramathibodi Hospital, a university‐affiliated tertiary care facility in Bangkok, Thailand, 682 patients were seen at the ED for acute severe hypertension. Of these, 298 patients were treated with antihypertensive drugs, primarily hydralazine (58.39%) and captopril (12.42%), with 26.51% receiving combination therapies. Significantly larger reductions in SBP of 36.38 ± 26.08 mmHg (vs. 30.90 ± 26.80) and in DBP of 15.34 ± 14.93 mmHg (vs. 11.33 ± 14.39) were noted among those given antihypertensive agents. Oral hypertensive agents conferred no advantage in reducing the risk of 1‐day (OR = 0.58, 95% CI = 0.26–1.27) and 7‐day ED revisits (OR = 1.11, 95% CI = 0.77–1.61), nor did they affect ED length of stay or BP control within 2 weeks. There were no reported MACE on day 1. MACE within 7 days in those not treated and treated with antihypertensive medications was similar, albeit inconclusive (OR 2.26, 95% CI 0.23–21.90) [22].

#### 4.9.2 Consensus Panel Synthesis

There was a lack of controlled clinical trials comparing gradual BP reduction over 24 to 48 h versus rapid BP reduction within minutes to hours among patients with acute severe hypertension. Observational studies provided low certainty evidence that there is no benefit from lowering BP rapidly among patients with acute severe hypertension. In contrast, possible harms of rapid BP lowering, such as watershed infarcts, have been described. The TF also considered the influence of institutional policies, legal considerations concerning the severity of complications of severe hypertension, logistical difficulties of early follow‐up in geographically isolated and depressed areas, and patient values and biopsychosocial experience on the decision to rapidly lower BP in patients. Given these considerations, the TF recommends a gradual reduction of BP over 24 to 48 h over immediate lowering to normal levels to reduce all‐cause mortality, MACE, and ED re‐visits.

Regardless of the decision to rapidly lower BP or not, there must be emphasis on patient education to appease the patient's emotional response and enhance understanding of the illness. The key role of non‐pharmacologic interventions, such as lifestyle modification in overall disease management and promotion of patient empowerment, must be highlighted.

## 5 Hypertensive Emergency

### 5.1 Clinical Question 6

Among patients with hypertensive emergency, what is the effect of rapid lowering (within 1 h) of blood pressure in reducing cardiovascular death, stroke, and MI?

### 5.2 Statement 6

- Among adult patients with hypertensive emergency and intracerebral hemorrhage (ICH), we suggest rapid BP lowering to SBP < 140 mmHg within 1 h to prevent worsening of intracerebral hemorrhage (ICH).
  (certainty of evidence: low; strength of recommendation: weak)

#### 5.2.1 Evidence From Literature

Based on 3 RCTs on patients with ICH, rapid control of elevated BP did not lead to a statistically significant difference in all‐cause mortality compared to conservative care (RR 0.90, 95% CI 0.81–1.01). In terms of neurologic deterioration or worsening of stroke, three studies showed that rapid control of BP significantly reduced the number of episodes of deterioration than those in the control group (RR 0.89, 95% CI 0.84–0.95) [36, 37, 38]. In terms of other outcomes, two RCTs measured quality of life through the EQ‐5D. There was no significant difference in terms of mobility (RR 0.86, 95% CI 0.73–1.02), self‐care (RR 0.90, 95% CI 0.77–1.03), and usual activities (RR 0.91, 95% CI 0.71–1.04). Rapid BP control showed significant improvement in terms of pain/ discomfort (RR 0.80, 95% CI 0.67–0.93) and anxiety/ depression (RR 0.83, 95% CI 0.68–0.98) [37, 39]. Rapid BP lowering showed significantly lower hematoma expansion compared to the control group (mean difference 2.8, 95% CI 1.04–4.56, *p* = 0.002) but no difference in terms of increase in edema volumes (mean difference 2.38, 95% CI −0.45 to 5.22, *p* = 0.10) [36]. There was no significant difference in terms of duration of hospital stay (IQR 12–35 vs. 11–33 days, *p* = 0.43) [37] Another study used the proportion of patients who were discharged after 7 days, which was significantly higher in patients with rapid BP lowering (OR 0.72, 95% CI 0.53–0.98, *p* = 0.03) [39].

In terms of safety, three RCTs had overall adverse events as an outcome, and those in the rapid BP control group had statistically significantly lower events (RR 0.79, 95% CI 0.73–0.85). Myocardial infarction was listed in these studies as an adverse event, and there were six cases in the treatment group and seven in the control group in three studies, showing no statistical difference between groups (RR 0.86, 95% CI 0.29–2.55).

#### 5.2.2 Consensus Panel Synthesis

Among patients with ICH, low‐certainty evidence found that rapid BP lowering had significant benefits in terms of reducing neurologic deterioration, hematoma volume expansion, and adverse events. However, there was no significant effect in terms of reducing rates of MI and cardiovascular death. Given this narrow scope of available evidence covering only RCTs among patients with acute ICH, the TF suggests BP lowering to SBP < 140 mmHg within 1 h specifically for patients suffering from ICH. For other cases of hypertensive emergency without ICH, there was a lack of direct evidence on the benefit of rapid BP lowering; hence, clear recommendations could not be made.

### 5.3 Best Practice Statement 3

Among adult patients with hypertensive emergency and complications other than ICH, we suggest rapid BP control to lower SBP as indicated in the table below.

### 5.4 Immediate: Within Minutes; Several Hours: 2 to 6 h

There is known heterogeneity of clinical characteristics among patients with hypertensive emergency, which may lead to variability in response, with some conditions benefiting more from rapid reduction, such as acute cardiogenic pulmonary edema and aortic dissection. BP reduction targets must be individualized for other conditions since the target of SBP < 140 mmHg was tested only for patients with acute ICH. An existing statement from the ISH [40] provides expert opinions on these targets; hence, it was adapted as a best practice statement after agreement by the CP (see Table 1).

**TABLE 1:** Individualized targets and timeframes for BP lowering in hypertensive emergency. (Adapted from: 2020 International Society of Hypertension global hypertension practice guidelines).

Clinical presentation	Timeframe and target BP	First‐line treatment
Malignant hypertension with or without TMA or acute renal failure	Several hours, MAP ‐20% to −25%	Nicardipine (first line) Labetalol (if available)
Hypertensive encephalopathy	Immediate, MAP −20% to −25%	Nicardipine (first line) Labetalol (if available)
Acute ischemic stroke and BP > 220 mmHg systolic or > 120 mmHg diastolic	1 h, MAP −15%	Nicardipine (first line) Labetalol (if available)
Acute ischemic stroke with indication for thrombolytic therapy and BP > 185 mmHg systolic or > 110 mmHg diastolic	1 h, MAP −15%	Nicardipine (first line) Labetalol (if available)
Acute coronary event	Immediate, systolic BP < 140 mmHg	Nitroglycerine (first line) Labetalol (if available)
Acute cardiogenic pulmonary oedema	Immediate, systolic BP < 140 mmHg	Nitroprusside or nitroglycerine (with loop diuretic)
Acute aortic disease	Immediate, systolic BP < 120 mmHg and heart rate < 60 bpm	Esmolol and nitroprusside or nitroglycerine or nicardipine
Eclampsia and severe pre‐eclampsia/HELLP	Immediate, systolic BP < 160 mmHg and diastolic BP < 105 mmHg	Labetalol or nicardipine and magnesium sulfate

Abbreviations: BP, blood pressure; HELLP, hemolysis, elevated liver enzymes and low platelets; TMA, thrombotic microangiopathy.

### 5.5 Clinical Question 7

Among patients with hypertensive emergencies, what is the effect of IV nitrates in reducing blood pressure and other cardiovascular clinical outcomes (mortality, stroke, and MI)?

### 5.6 Statement 7

- Among adult patients with hypertensive emergency and a cardiovascular event*, we recommend the use of IV nitrates to lower BP and reduce all‐cause mortality.
  (certainty of evidence: low, strength of recommendation: strong)
  *acute coronary syndrome, acute cardiogenic pulmonary edema

#### 5.6.1 Evidence in Literature

In a meta‐analysis of 65 RCTs assessing all‐cause mortality among patients with acute coronary events, nitrates had a pooled statistically significant reduction in all‐cause mortality (RR 0.81, 95% CI [0.74–0.89], *p* < 0.0001) if given as immediate treatment, defined as started within 24 h of onset and lasting for a maximum 2 days (6 trials, *n* = 82,624). For short‐term treatment started within 24 h of onset and lasting for a maximum of 10 days, only 6 trials out of the 18 trials (*n* = 78,178) on nitrates found a statistically significant reduction in all‐cause mortality (RR 0.91, 95% CI [0.86–0.97], *p* = 0.003. At durations lasting > 10 and > 30 days, nitrates were not associated with a difference in all‐cause mortality. The authors concluded that nitrates administered within 24 h of symptom onset significantly decreased all‐cause mortality at 2 days (4 to 8 deaths prevented per 1000); however, there was no added benefit of continuing nitrates beyond day 2. In terms of BP reduction, nitrates had a statistically significant reduction in both SBP (WMD −12.67 mmHg, 95% CI −14.51, −10.83) and DBP (WMD −7.50 mmHg, 95% CI −9.07, −5.93) compared to no treatment or placebo during the first 24 h of an acute myocardial infarction. It is, however, important to note that high BP at baseline was not part of the inclusion criteria in any of the studies included in the meta‐analysis. The overall weighted mean BP at baseline (for the included studies) was 133/82.2 mmHg for those randomized to an active drug and 133/82.7 mmHg for those included in the placebo or no treatment group [12].

For patients with acute cardiogenic pulmonary edema, a retrospective cohort study of 8,863 Japanese patients presenting with a primary diagnosis of acute heart failure showed that vasodilator use was not associated with reduced in‐hospital mortality (7.5% vs. 8.8% in patients without vasodilators; *p* = 0.098) nor was it associated with a difference in length of ICU/CCU (6.2 days vs. 5.8 days in patients without vasodilators) and hospital stays (23.2 days vs. 23.9 days in patients without vasodilators). In the same study, subset analysis showed that reductions in hospital mortality were observed with vasodilator use among patients with SBP greater than 180 mmHg (OR 0.25, 95% CI 0.10–0.75) and those who did not present with atrial fibrillation on electrocardiogram on admission (OR 0.70, 95% CI 0.60–0.90). The reduction in mortality was not clinically significant in those who presented with severe pulmonary congestion (OR 0.79, 95 CI 0.60–1.02). In the same study, nitrates (nitroglycerin and isosorbide dinitrate compared to carperitide) were associated with better outcomes, including in‐hospital mortality rates (5.1% vs. 6.7% in patients with carperitide use, *p* = 0.041) as well as length of ICU/CCU stay (4.8 days vs. 6.1 days in patients with carperitide use; *p* < 0.0001) and length of hospital stay (21.2 days vs. 24.3 days in patients with carperitide use; *p* < 0.0001) [41].

Another study examined the association between diuretics, nitrates, and opiates and 7‐day mortality among patients presenting with acute cardiogenic pulmonary edema. Results showed that nitrates had lower mortality rates compared to opiates and diuretics; however, after adjustment for confounding factors, there was no evidence of any association between treatment with nitrates and 7‐day mortality [42]. The NITRO‐EAHFE Study, a prospective, multicenter cohort study in Spain, also concluded that IV nitrates did not influence early mortality or new visits in patients with acute heart failure. A separate subgroup analysis of patients with hypertensive acute pulmonary edema also found no significant difference in mortality between groups (nitrates group vs. control group), but findings may be influenced by the small number of patients analyzed [43].

#### 5.6.2 Consensus Panel Synthesis

There is a lack of direct evidence on the effect of IV nitrates in reducing BP and other CV outcomes in patients with hypertensive emergency per se. The majority of studies on the benefit of IV nitrates were performed among patients with acute coronary events and acute cardiogenic pulmonary edema, and while these conditions often present as hypertensive emergencies, the evidence was indirect. IV nitrates significantly reduced mortality among patients with acute coronary events, while some studies showed that nitrates may also decrease all‐cause mortality in patients with acute cardiogenic pulmonary edema. IV nitrates are relatively low‐cost and widely available compared to other IV antihypertensives. It is also important to consider the variable and potentially harmful effects of IV nitrates for specific patient groups with hypertensive emergencies, such as patients with ICH, where its use may be inappropriate. Given these considerations, the TF strongly recommends the use of IV nitrates only for patients presenting with both a hypertensive emergency and a cardiac event.

### 5.7 Clinical Question 8

Among patients with hypertensive emergencies, what is the effect of IV nicardipine in reducing blood pressure and other cardiovascular clinical outcomes (mortality, stroke, and MI)?

### 5.8 Statement 8

- Among adult patients with hypertensive emergency, we recommend the use of IV nicardipine to lower BP and reduce hospital length of stay.
  (certainty of evidence: low, strength of recommendation: strong)

#### 5.8.1 Evidence From Literature

A systematic review by Peacock et al., which included 10 studies among different populations with hypertensive crises (total population *n* = 990, with 477 in the nicardipine group and 513 in the labetalol group), showed that nicardipine and labetalol had similar efficacy in reducing BP. Among patients with non‐traumatic ICH and a MAP of 120–160 mmHg, nicardipine had a mean MAP reduction of −23.4 mmHg (−15.6%) while labetalol had a mean MAP reduction of −24.1 mmHg (−17.8%). Nicardipine was noted to have less variability in MAP values (nicardipine 8.19 mmHg, labetalol 10.78% *p* = 0.003), with fewer dosage adjustments needed (nicardipine 2, labetalol 4 *p* < 0.001), and less need for additional agents to control the BP (nicardipine 8%, labetalol 33%, *p* = 0.013). Moreover, the nicardipine group was able to achieve target BP earlier, within 1 h, among patients with ICH (nicardipine 33%, labetalol 6%, *p* < 0.02). Nicardipine also had a greater portion of time spent at goal BP in a span of 24 h (nicardipine 82.5%, labetalol 48.5%, *p* < 0.001), with a shorter mean time to goal BP (nicardipine 30 min, labetalol 90 min, *p* = 0.026). Patients on nicardipine were found to have shorter hospital length of stay compared to labetalol (nicardipine 4 days, labetalol 7 days, *p* = 0.01) [44]. The findings from the 2012 systematic review were supported by a succeeding RCT in 2014, which noted that patients administered nicardipine achieved the target range more frequently compared to those given labetalol (92% vs. 78%, *p* = 0.046). In terms of systolic blood pressure (SBP) measures, nicardipine‐treated patients reached the target range more than those treated with labetalol (46% vs. 25%, *p* = 0.024). Labetalol recipients showed a higher likelihood of needing rescue medications to aid with blood pressure control (27% vs. 17%, *p* = 0.020) [45].

A more recent systematic review and meta‐analysis done in 2022, comparing nicardipine and labetalol, which included 5 retrospective cohorts and 1 prospective cohort, had similar findings of nicardipine's superiority over labetalol in terms of time at goal BP among stroke patients (CI: 0.112–0.438, *p* = 0.001). However, nicardipine was found to have higher adverse events, including hypotension and tachycardia (CI: 1.077–2.113, *p* = 0.017). However, the studies had some concerns for bias, particularly in population selection, performance, and detection bias [46]. Another safety study by Kim et al. in 2012, aiming to assess the safety of nicardipine among 52 study participants, noted complications during nicardipine infusion, including hypotension in 1 patient (1.9%) and findings of tachycardia or an arrhythmia in 4 patients (7.7%). No patient was noted to have bleeding or renal dysfunction [47].

Few studies compared IV nicardipine with other antihypertensives. One RCT by Yang et al. comparing nicardipine and nitroprusside for ED patients with hypertensive crisis and acute pulmonary edema found that both groups saw notable drops in their SBP and DBP post‐treatment, with no substantial differences observed over time between the groups [48]. Another study by Seifi et al. in 2023, comparing clevidipine with nicardipine, showed no statistically significant difference in BP effect; however, clevidipine reached the target SBP 23 min faster than nicardipine, with less total volume infused to achieve target BP. Notably, clevidipine is not available in the Philippines as of this writing [49].

In terms of safety, both nicardipine and labetalol were safe in hypertension in pregnancy, with no significant difference in the number of patients achieving target BP (nicardipine 70%, labetalol 63%, *p* = 0.58) and similar time to reach target BP (nicardipine 11.1 min, labetalol 12.4 min, not significant) [44]. This was concurrent with a later network meta‐analysis comparing different drugs for severe hypertension in pregnancy, which showed no significant differences in the number of patients achieving target BP among nicardipine, diazoxide, nifedipine, and glyceryl trinitrate. However, nicardipine required significantly more doses than nifedipine [50, 51].

#### 5.8.2 Consensus Panel Synthesis

Studies comparing IV nicardipine with IV labetalol among patients with different conditions (stroke, critical care, surgery, pediatrics) showed comparable efficacy and safety in BP control, but nicardipine had a more predictable and consistent control of BP than labetalol, with less requirement for additional antihypertensive agents to achieve the target. IV nicardipine demonstrated safety even among pregnant women and populations with chronic kidney disease. Furthermore, existing recommendations from other professional groups recommend IV nicardipine as the first‐line drug for hypertensive emergencies. Currently, IV nicardipine has widespread availability and affordability in community and hospital settings due to the inclusion of IV nicardipine in the Philippine National Drug Formulary. Due to the above considerations, the TF strongly recommends IV nicardipine as the first‐line drug to lower BP in a hypertensive emergency.

### 5.9 Clinical Question 9

Among patients with hypertensive emergencies, what is the effect of IV labetalol in reducing blood pressure and other cardiovascular clinical outcomes (mortality, stroke, and MI)?

### 5.10 Statement 9

- Among adult patients with hypertensive emergency, we suggest not to use IV labetalol for reducing BP.
  (certainty of evidence: very low, strength of recommendation: weak)

#### 5.10.1 Evidence in Literature

A systematic review of six retrospective/prospective cohort studies evaluated the safety and efficacy of IV labetalol compared to IV nicardipine in the management of acute stroke, including ICH, acute ischemic stroke, and subarachnoid hemorrhage. There was a total of 3443 patients in this study, with 3219 in the IV nicardipine group and 224 in the IV labetalol group. Time to reach target BP was significantly better for IV nicardipine (0.275 SMD, 95 CI, 0.112–0.438, *p* = 0.001). However, the endpoint of superior time to reach goal BP has not been shown to directly affect cardiovascular morbidity and mortality among acute stroke patients. In terms of safety, there was a higher incidence of adverse events for the nicardipine group, particularly for tachycardia (OR 1.509, 95% CI, 1.077–2.113, I2 = 0.00%, *p* = 0.017) [46].

#### 5.10.2 Consensus Panel Synthesis

There was a lack of controlled clinical trials, with only nonrandomized studies exploring the comparison of IV labetalol and IV nicardipine for patients with acute stroke. Compared to IV nicardipine, IV labetalol use led to a smaller magnitude and slower rate of BP reduction, with no significant difference in terms of CV outcomes. In addition, there is limited availability and relatively higher cost of IV labetalol in most community and hospital settings due to its non‐inclusion in the Philippine National Drug Formulary. Due to these considerations, the TF suggests not using IV labetalol routinely among patients with hypertensive emergencies.

### 5.11 Clinical Question 10

Among patients with hypertensive emergencies, what is the effect of IV hydralazine in reducing blood pressure and other cardiovascular clinical outcomes (mortality, stroke, and MI)?

### 5.12 Statement 10

- Among non‐pregnant adult patients with hypertensive emergency, we suggest not to use IV hydralazine for reducing BP.
  (certainty of evidence: low, strength of recommendation: weak)

#### 5.12.1 Evidence in Literature

There were no RCTs evaluating the BP reduction and MACE in patients with hypertensive emergencies given IV hydralazine in hospital settings. A prospective observational study included 94 hospitalized patients with an order for IV hydralazine (mean age 69 ± 18 years, 48% women, 89% with chronic hypertension). Only 4 (2%) of patients had evidence of an urgent hypertensive condition. Changes from baseline BP correlated with baseline BP, such that there were smaller changes in BP for those with lower baseline BP (−3 ± 20 mmHg) compared to those with higher baseline BP (−35 ± 25 mmHg). Eighty‐three percent of patients who received hydralazine failed to achieve a > 25% reduction in BP after the initial 2 h treatment period [52].

In terms of safety, hydralazine is inconsistent in its effects and difficult to titrate. Unexpected, sudden drops in BP beyond intended targets limit its use as a treatment in the ED and hospital setting [10, 53]. The resultant tachycardia from IV hydralazine also limits its use in patients, as seen in a retrospective cohort of non‐critically ill individuals [54].

#### 5.12.2 Consensus Panel Synthesis

There was a lack of RCTs on the use of IV hydralazine among non‐pregnant adults. Low‐certainty observational studies showed a lack of efficacy for reducing BP or hospital LOS, with a greater incidence of AEs. Recommendations from other professional groups also relegate hydralazine as a last‐line drug for resistant hypertension. Due to these considerations, the TF suggests against the use of IV hydralazine routinely among patients with hypertensive emergencies. However, the TF considered the relatively affordable cost of IV hydralazine relative to other IV antihypertensives and the limited availability of other more efficacious IV antihypertensives in geographically isolated areas, which may justify its use in special circumstances.

### 5.13 Clinical Question 11

Among patients with hypertensive emergencies, what drugs can be given in the primary care setting while the patient is being transferred to a hospital?

### 5.14 Statement 11

- Among adult patients with hypertensive emergency in the primary care setting, while the patient is being transferred, we suggest the use of antihypertensive medications to reduce BP. IV antihypertensives are preferred, but if not available, oral antihypertensives may be used.
  (certainty of evidence: very low, strength of recommendation: weak)

#### 5.14.1 Evidence in Literature

The review yielded no RCTs or meta‐analyses that directly evaluated the use of antihypertensive medications in hypertensive emergencies in the primary care setting. An observational study by Merlo et al. evaluated the management strategy of general practitioners (GPs) for 164 patients with severely elevated BP. Most or 99 (65%) of the patients were asymptomatic, 50 (31%) had acute severe hypertension, and only 15 (9%) had hypertensive emergency. Results showed there was no difference in the occurrence of cardiovascular events in patients who were asymptomatic, in hypertensive urgency, or hypertensive emergency during presentation [55].

A Cochrane review included 15 RCTs from 1983 to 2004, totaling 869 participants. Although BP entry criteria differed between included trials, all participants presented with acute end‐organ damage. The antihypertensive drugs used were nitrates (9 trials), ACEi (7 trials), CCBs (6 trials), peripheral alpha‐1 blockers (4 trials), diuretics (3 trials), direct vasodilators (2 trials), and dopamine agonists (1 trial). For all‐cause mortality, the studies could not be pooled due to heterogeneity in the outcomes. There were 7 trials that evaluated mortality, but only 3 RCTs reported actual deaths, which totaled 6, while the 4 RCTs reported no deaths in their study, with a follow‐up that ranged from 6 to 24 h. The review was also unable to pool the outcome of composite non‐fatal cardiovascular events but reported the rates of MI and pulmonary edema requiring mechanical ventilation in five studies. One placebo‐controlled trial and three other antihypertensive drugs head‐to‐head trials showed there was no statistical difference in MI rates between these interventions. Similarly, there was no statistically significant difference between placebo and captopril (RR 0.40, 95% CI 0.09 to 1.86), nitrates and alpha‐adrenergic antagonist (RR 4.12, 95% CI 0.20–84.24), or nitrates and ACE‐i (RR 0.33, 95% CI 0.01 to 7.78) [12].

The results of the pooled analysis from three trials showed that there was no statistical difference in reductions in SBP (WMD ‐5.62 mmHg, 95% CI −13.26 to 2.02) or DBP (WMD −3.36 mmHg, 95% CI −8.7 to 1.98) between nitrates (nitroglycerin or isosorbide) and furosemide. Two studies, one using nitroprusside and the second one using nitroglycerin, compared the BP‐lowering effect of these nitrates to an alpha‐1 antagonist, urapidil. There was also no statistical difference between the reductions in SBP (WMD ‐3.49 mmHg, 95% CI −9.31 to 1.43) and DBP (WMD 0.76 mmHg, 95% CI −2.7 to 4.23) between these groups. In the trial comparing nitrates with a dopamine agonist, there was a statistically greater reduction in SBP (WMD −14.00 mmHg, 95% CI −27.72 to 0.28) but not in DBP (WMD −2.00 mmHg, 95% −11.74 to 7.74). There was also no statistically significant BP reduction between nitrates and ACEi (SBP WMD 3.33 mmHg, 95% −7.37 to 14.03, DBP WMD −0.33 mmHg, 95% CI −6.69 to 6.03), nitrates and CCB (SBP WMD 4.67 mmHg, 95% CI −3.97 to 13.32, DBP WMD 3.5 mmHg, 95% CI −3.94 to 10.94), and nitrates and hydralazine (SBP WMD −3.67 mmHg, 95% CI −20.41 to 13.07, DBP WMD 3.34 mmHg, 95% CI −4.82 to 11.5). The pooled data from four trials comparing ACEi with a CCB showed a significant DBP reduction (WMD −7.86 mmHg, 95% CI 4.92 to 10.81) but no significant reduction in SBP (WMD −1.68 mmHg, 95% CI 4.92 to 10.81). ACEi was found to statistically lower SBP (WMD −20 mmHg, 95% CI −22.85 to −17.39) and DBP (WMD −3.70 mmHg, 95% CI −7.08 to −0.31) compared to the alpha‐1 adrenergic antagonist. The trial comparing alpha‐blocker with nitroglycerine was the only trial that reported withdrawal due to adverse events (5% vs. 2.7%; [RR 3.38, 95% CI 0.17–68.84]), which was found to be not statistically significant [12].

#### 5.14.2 Consensus Panel Synthesis

There was very‐low‐certainty evidence on the use of antihypertensives for hypertensive emergencies in the primary care setting due to indirectness, imprecision, nonreporting of effect size, and the uncontrolled nature of available studies. Given the benefits of rapid BP lowering and the dangers of prolonging an untreated hypertensive emergency, the TF suggests the use of IV antihypertensives over oral hypertensives. However, recognizing the limited availability and accessibility of IV antihypertensives in isolated areas as well as the relative difficulty of securing an IV access, oral antihypertensives may be used should IV medications be unavailable.

### 5.15 Best Practice Statement 4

- Among adults with hypertensive emergency in the primary care setting, while the patient is being transferred, we suggest referral to a health facility capable of monitoring and safely lowering BP.

Patients with hypertensive emergencies will benefit from close monitoring and rapid lowering of BP; hence, transfer to a facility capable of monitoring BP, administering IV antihypertensives, and addressing adverse events is required. The TF discussed the importance of establishing linkages with the public health care provider network and local referral system to facilitate such transfers.

**FIGURE 1:**
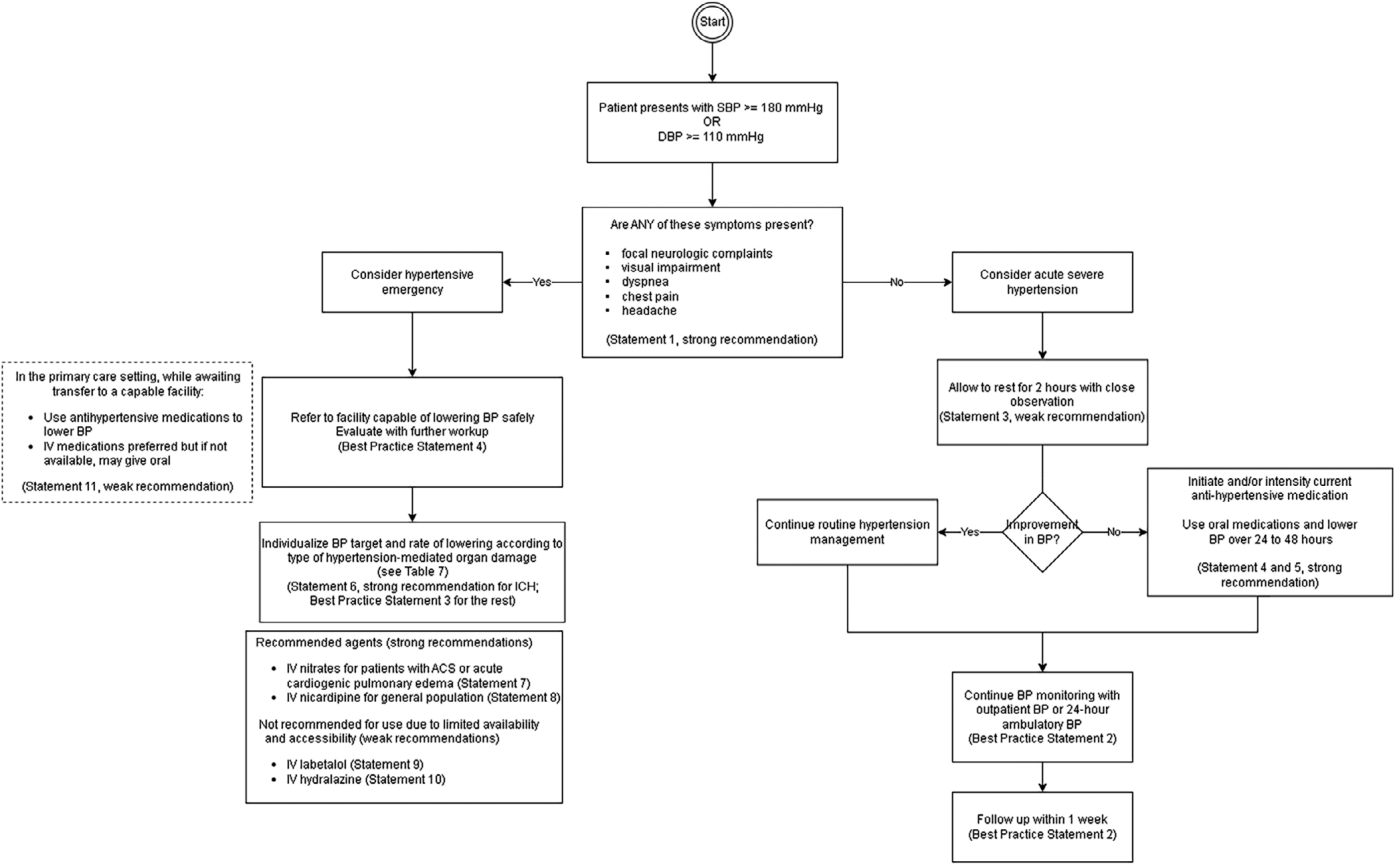
Algorithm based on the above recommendations, developed by the authors for this study.

Integrating the recommendations of this guideline, the authors formulated an algorithm for the diagnosis and management of acute severe elevations in BP (see Figure 1).

## 6 Limitations of the Guidelines

The strength of recommendations in this guideline was limited by the lack of direct evidence on specific aspects of severe BP elevation management. For the majority of the medications assessed, the focus of existing trials was on the degree and rate of BP reduction, with less emphasis on CV outcomes such as MI, stroke, or MACE. Furthermore, there were limited head‐to‐head comparisons among different medications, precluding any decisions on the relative efficacy of one agent compared to another. The impact of different treatment approaches on CV outcomes among patients with severe BP elevation remains an active research gap, which requires the attention of international and local researchers.

On the topic of workflows, there was limited to no literature on the impact of specific referral pathways on the outcomes of patients with severe BP elevation, whether for hypertensive emergency or otherwise. Due to this, critical recommendations on referral approaches and management while in transition from primary care to ED were based on expert opinion only. The dearth of literature in the implementation aspect of these management approaches should be the topic of further investigation.

The majority of the evidence reviewed was among Western populations, and most studies performed in Asia were performed among East Asians. Differences in pharmacodynamics, pharmacokinetics, and epigenetics among Asian populations may give rise to variability in BP response and outcomes; hence, additional studies among Filipinos are required to generate direct evidence. Furthermore, economic analyses and studies on patient‐centered outcomes, such as patient‐reported symptom measures and quality of life, were also limited for patients with severe BP elevation, necessitating further research.

## 7 Conclusion

This guideline is intended for use by general practitioners, allied health professionals, emergency medical personnel, specialists, and any healthcare worker who may encounter adult patients with hypertension. It presents a set of contextualized recommendations and best practice statements guided by systematic reviews of evidence and consensus panel discussions. These recommendations aim to address key clinical questions on the diagnosis and management of severe BP elevation with the end goal of improving individual and public health outcomes.

The TF calls on the reader to consider equity, applicability, and individualization when applying the recommendations of this CPG in medical practice. Comprehensive history taking, physical examination, and judicious monitoring are fundamental to patient assessment, and the insights gathered from these core processes should guide the overall management.

## Funding

This CPG was prepared by the Philippine Society of Hypertension (PSH), Philippine Heart Association (PHA), Philippine College of Emergency Medicine (PCEM), and Philippine Academy of Family Physicians (PAFP). Funding for this CPG was made possible by the Philippine Society of Hypertension and the Philippine Heart Association.

## Conflicts of Interest

**Committee**: Aileen Espina, MD, Marcellus Ramirez, MD, Josephine Sanchez, MD

## Consensus Panel Members

Philippine College of Emergency Medicine: Dave Gamboa, MD, Romulo Babasa, MD

Philippine Academy of Family Physicians: Suzanne Langcauon, MD, Krizia Tesorio‐Mier, MD

Philippine Society of Hypertension: Alberto Atilano, MD

Philippine Heart Association: Marc Denver Tiongson, MD, Karen De Leon, MD

Philippine Medical Association: Minerva Calimag, MD

Municipal Health Office: Daystar Sedillo, MD

Patient Advocate: Ramoncito Herrera

Philippine Nurses Association (PNA): Jan Vincent Cabasag, RN

Department of Health (non‐voting observers): Sean Paolo Ohrelle Aquino, MD, Ruth Divine Agustin, MD
